# The Efficacy of Aprotinin Combinations with Selected Antiviral Drugs in Mouse Models of Influenza Pneumonia and Coronavirus Infection Caused by SARS-CoV-2

**DOI:** 10.3390/molecules27154975

**Published:** 2022-08-05

**Authors:** Andrey A. Ivashchenko, Bogdan A. Zagribelnyy, Yan A. Ivanenkov, Ilya A. Ivashchenko, Ruben N. Karapetian, Dmitry V. Kravchenko, Nikolay P. Savchuk, Elena V. Yakubova, Alexandre V. Ivachtchenko

**Affiliations:** 1 ChemDiv Inc., San Diego, CA 92130, USA; 2 Viriom Inc., San Diego, CA 92130, USA; 3 ASAVI LLC, 1835 E. Hallandale Beach Blvd, #442, Hallandale Beach, FL 33009, USA

**Keywords:** aprotinin, antiviral drugs, SARS-CoV-2, COVID-19, influenza

## Abstract

The efficacy of aprotinin combinations with selected antiviral-drugs treatment of influenza virus and coronavirus (SARS-CoV-2) infection was studied in mice models of influenza pneumonia and COVID-19. The high efficacy of the combinations in reducing virus titer in lungs and body weight loss and in increasing the survival rate were demonstrated. This preclinical study can be considered a confirmatory step before introducing the combinations into clinical assessment.

## 1. Introduction

In December 2019 in Wuhan city, Hubei province, China, a new coronavirus was first identified, causing coronavirus disease 2019 (COVID-19), then called SARS-CoV-2 due to the similar symptoms to SARS from the coronavirus outbreak in 2003. On 11 March 2020, the World Health Organization (WHO) declared the COVID-19 pandemic because of the extraordinary threat to public health worldwide [1]. As of 7 June 2022, 536,108,378 confirmed cases of people infected with COVID-19 were registered in the world, of which 6,321,883 deaths, including 86,637,487 confirmed cases and 1,033,830 deaths, were in the USA [2]. 

**Figure 1:**
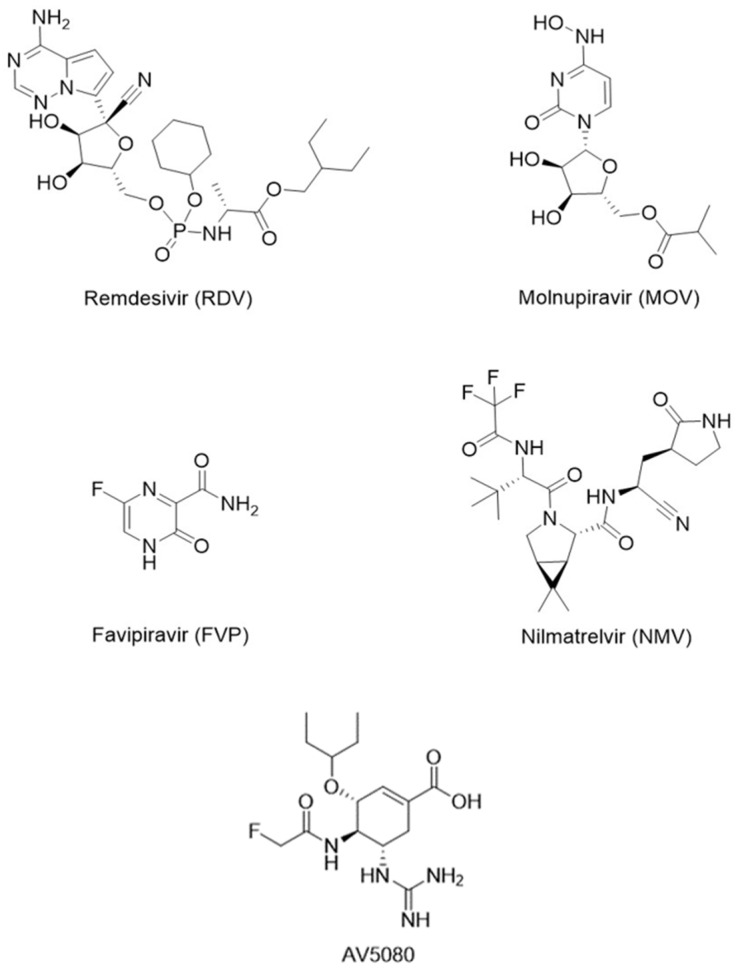
Structural formulas of registered antiviral drugs for COVID-19 treatment and anti-influenza drug candidate AV5080.

To suppress the COVID-19 pandemic, various treatment or prevention strategies are used: vaccination, the repurposing of already-known antiviral drugs, and the search for new active molecules for the identified molecular targets of SARS-CoV-2. Small molecules remdesivir (RDV), molnupiravir (MOV), nirmatrelvir (NMV), and favipiravir (FVP) (Figure 1) have emergency use authorization for the treatment of coronavirus disease in different countries. However, several studies on the effectiveness of these drugs in monotherapy are highly controversial [3,4]. These drugs are practically ineffective in the treatment of moderate, severe, especially severe, and critical COVID-19 patients. They can be only recommended for mild-to-moderate COVID-19 patients. 

Influenza affects about 10% of the world’s population every year. The most common complications of influenza include viral or bacterial pneumonia, which kills about half a million people each year [5]. The Centers for Disease Control and Prevention (CDC) estimates that flu has resulted in 29–41 million illnesses, 380,000–710,000 hospitalizations, and 22,000–38,000 deaths annually between 2010 and 2020 [6,7].

Currently, the main anti-influenza drugs used are neuraminidase (NA) inhibitors: oral oseltamivir, inhaled zanamivir, and intravenous peramivir. Adamantanes are not recommended for the treatment of influenza due to the high resistance of influenza A to these drugs [8]. Treatment with NA drugs is effective if started within a few days of the onset of flu symptoms. In addition, the rapid emergence of resistant strains to known NA drugs and new resistant influenza strains remains a serious problem. Therefore, the development of new anti-influenza drugs and new therapies for influenza pneumonia remains an urgent task. Such a drug could be an analogue of oseltamivir, AV5080 (Figure 1), which showed increased antiviral activity against several influenza strains, including oseltamivir-resistant strains [9,10,11]. 

Recent studies have shown that aprotinin (APR) is able to inhibit the replication of SARS-CoV-2 [12]. APR is a natural protease inhibitor, with a long history of clinical use since the 1960s and a good safety profile [13]. Initially, APR was used to treat acute pancreatitis, then complex surgical interventions, such as heart and liver surgeries [14,15,16]. Since the early 1980s, APR has been actively studied as an anti-influenza drug [17,18,19,20,21], and, in recent years, protease inhibitors, including APR, have been actively studied as anti-coronavirus drugs [12,22,23]. APR is a nonspecific inhibitor that competitively and reversibly inhibits the activity of several different serine proteases, especially trypsin, chymotrypsin, plasmin, and kallikrein. The inhibition of kallikrein leads to the inhibition of factor XIIa formation, the inhibition of the intrinsic coagulation pathway, fibrinolysis, thrombin generation, and the attenuation of the pro-inflammatory response. 

APR inhibits transmembrane serine protease 2 (TMPRSS2), which is actively involved in the viral entry process [12,24,25,26,27,28,29], and also the cleavage of hemagglutinin (HA), which is required for influenza virus fusion with a host cell and which can also be facilitated by TMPRSS2 during viral egress and by the membrane-bound human airway-trypsin like protease (HAT) prior to attachment to host cells [19,30,31,32,33,34,35].

Previous studies have shown that APR inhibits a broad spectrum of influenza A viruses (IAV) and influenza B viruses (IBV). Anti-influenza viral activity includes seasonal human IAVs (H1N1 and H3N2 subtypes): A/CA/04/09 (H1N1) [34], A/PR/8/34 (H1N1) [18,35,36], A/WSN/34 (H1N1) [37], A/CA/04/09 (pH1N1), A/Hamburg/05/2009 (pH1N1) [36], A/Aichi/2/68 (H3N2) [18,35,37], and A/PH/2/82 (H3N2) [35]; avian IAVs A/AB/Kor/CN2/09 (H5N2), A/AB/Kor/CN05/09 (H6N5) and A/Ck/Kor/01310/01 (H9N2) [35]; an oseltamivir-resistant IAV: A/Bris/10/07 H3N2 [37]; IBV B/Seoul/32/2011 [35], B/Hong Kong/73 [30], and B/Lee/40 [30]. It should be noted that the aprotinin-based aerosol patented in 2010 for the treatment of viral respiratory infections [38] has been approved in Russia for the treatment of influenza [19]. 

In our present study, we aimed to show the use of aprotinin (APR) in the combined treatment of viral infections, primarily caused by influenza A virus (H1N1) and SARS-CoV-2.

## 2. Results

### 2.1. Efficacy of the Combinational Use of Aprotinin (APR) Is Higher Than Selected Antiviral Drugs in a Model of Influenza Pneumonia in Mice Infected Intranasally with Influenza A/CA/04/2009 (H1N1)

**Figure 2:**
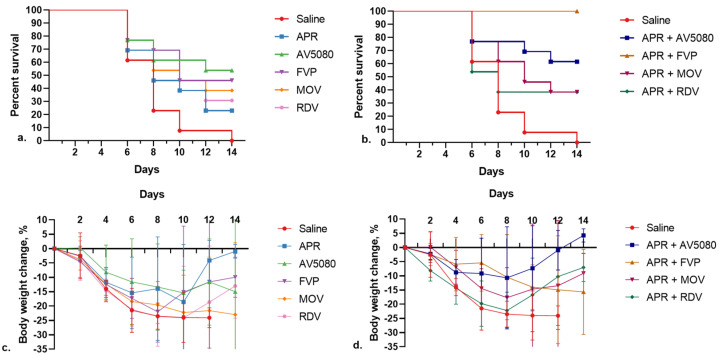
Survival (**a**,**b**) and change in the body weight (**c**,**d**) of mice treated with the drugs when infected with influenza virus A/California/04/2009 (H1N1).

The results are shown in Table 1 and in Figure 2, from which it follows that drugs combinations are significantly more effective than single components in terms of average life expectancy increase, dynamics of weight loss, titer value of the influenza virus in the mouse lungs 5 days after infection (lgTCID50/mL), and mortality.

### 2.2. Efficacy of the Combinatorial Use of Aprotinin (APR) Is Higher Than the Individual Use of APR and Selected Antiviral Drugs in a Model of Coronavirus Infection Caused by SARS-CoV-2 in Transgenic Mice

**Figure 3:**
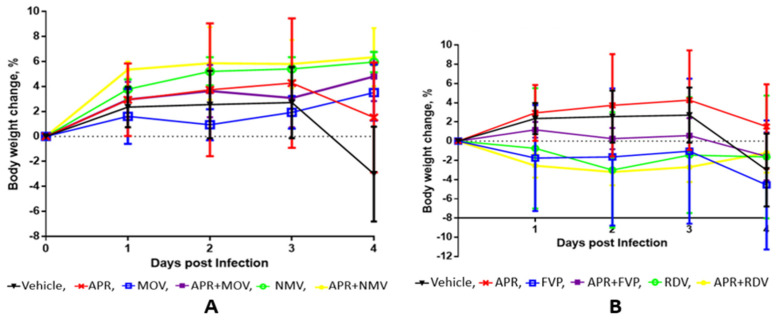
Weight change in drug-treated SARS-CoV-2 infected patients when used monotherapy (**A**) and combination therapy (**B**).

The results of the efficacy of the combinatorial use of APR and selected antiviral drugs in a model of coronavirus infection caused by SARS-CoV-2 in transgenic mice are shown in Table 2 and in Figure 3, from which it follows that the APR + MOV and APR + NMV combinations are more effective than single components in the ability to reduce the virus titer in lungs and the dynamics of body weight loss after 5 days, while the effectiveness of the APR + FVP and APR + RDV combinations is comparable with their constituent components.

## 3. Discussion

Given the unique therapeutic profile (anti-influenza, anti-inflammatory, and anti-thrombotic) of APR, we expected a synergistic and/or additive effect from the combined treatment including APR and selected antiviral drugs. As the latter, we used anti-influenza drugs AV5080 [11], FVP [39,40], MOV [41,42,43], and an anti-SARS-CoV-2 drug RDV [44,45], which has no activity against influenza viruses [46]. AV5080, FVP, MOV, and RDV were used as reference drugs, and saline was used as a control.

The efficacy data of the treatment with drug combinations of APR and the antiviral drugs in a model of influenza pneumonia in mice infected with IAV A/CA/04/2009 (H1N1) are presented in Table 1 and Figure 2.

In this study, we used combinations of APR + AV5080, APR + FVP, APR + MOV, and APR + RDV. The effectiveness of APR, AV5080, FVP, MOV, and RDV were studied in parallel as references, and saline was used as a control.

As can be seen from Table 1, all combinations of APR + AV5080, APR + FVP, APR + MOV, and APR + RDV provided a decrease in the virus titer in the lungs of mice 5 days after infection by 3.5–4.98 orders of magnitude compared with the control group and by 0.59–2.67 orders of magnitude compared with control groups treated with individual APR, AV5080, FVP, MOV, and RDV drugs included in combination treatments. These data indicate the high efficiency of combination therapy with APR and the selected antiviral drugs.

The average life-expectancy groups of animals receiving combined therapy was higher than in the control group and the corresponding comparison groups. For example, in monotherapy groups #1, #2, #3, #4, #5, and #6, it was 7.6, 10.7, 10.8, 11.4, 12.4, and 13.0 days, respectively, and in combination therapy groups #7, #8, #9, and #10, it was 10.4, 11.8, 16.0, and 14.0 days, respectively (see Table 1). An exception can be seen in the combined group APR + RDV No. 7, for which the average life expectancy was 10.4 days, which is slightly lower than in the comparison groups APR No. 2 (10.7 days) and RDV No. 3 (10.8 days).

In groups No. 10 and No. 9 of animals receiving combined therapy, compared with the control group No. 1 and comparison groups No. 2, No. 6, and No. 5, there was a lower mortality of animals (20%, 0%, 100%, 70%, 30%, and 40%, respectively). In groups No. 8 and No. 7 of animals receiving combined therapies APR + MOV and APR + RDV, compared with comparison groups No. 4 and No. 3, comparable animal mortality was observed (Table 1).

Coronavirus disease-2019 (COVID-19) is caused by SARS-CoV-2, which is a new member of the coronavirus family. Various antiviral drugs have been researched for COVID-19 treatment including RDV, MOV, FVP, and NMV [47]. 

The APR prophylactic treatment of healthcare personnel provided 93.3% protection. Only 2 out of 30 workers (6.7%) were infected with SARS-CoV-2. At the same time, it has been reported that the average level of infection among medical workers can reach 29% [48].

A prospective clinical study showed the high efficacy of APR in the inhaled and intravenous treatment of hospitalized patients with moderate pneumonia associated with COVID-19 [49]. The median time to the normalization of the elimination of SARS-CoV-2, body temperature, CRP, and D-dimer was 3 to 9 days (IQR = 2–9 days) [50]. Later, these results were convincingly confirmed by a full-fledged phase III randomized clinical trial evaluating the safety and efficacy of APR in the treatment of patients with moderate COVID-19 [51].

It should be noted that all reported clinical studies on the prevention and treatment of COVID-19 with APR administration were not accompanied by any adverse events or reactions [49,50]. 

Combination therapy with APR and inhibitors of the RNA virus is currently represented by only one prospective single-center clinical study, which examined the effectiveness of the combination of APR and FVP [5]. This combination therapy has been shown to be highly effective in the treatment of hospitalized patients with moderate COVID-19-related pneumonia. The median of time to the elimination of SARS-CoV-2, the normalization of CRP concentration, D-dimer level, and body temperature, as well as improvement in clinical status by 2 points on the WHO-OSCI, were from 1 to 5 days, while in comparison, cohorts 2, 3, and 4 had median values that varied from 3 to 11 days, 2 to 14 days, and 4 to 14 days, respectively (Table 2).

In this work, we studied the efficacy of combinations that include, along with APR, the well-known SARS-CoV-2 antiviral agents, including MOV, NMV, FVP, and RDV in a transgenic mouse model that uses the mouse-adapted SARS-CoV-2 strain.

**Figure 4:**
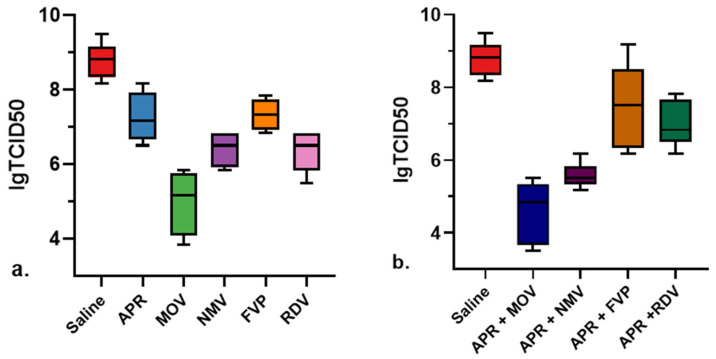
The titer value of the virus in the mouse lungs 4 days after infection (lgTCID50/mL) in a model of transgenic mice infected with the mouse-adapted SARS-CoV-2 strain when used monotherapy (**a**) and combination therapy (**b**).

As can be seen from Figure 4, in groups of mice that received combinations of APR + MOV or APR + NMV, the titer value of the virus in the mouse lungs of animals (TCID50/mL) 4 days after infection was 4.2 and 3.2 orders of magnitude lower than in the group of untreated animals. Compared to the comparison groups, groups APR and MOV, in the APR + MOV group, the lgTCID50/mL value was reduced by 2.72 and 0.43 units, respectively. In the APR + NMV group, compared to groups APR and NMV, gTCID50/mL was reduced by 1.68 and 0.85 orders, respectively.

In groups that were treated with combinations of APR + FVP and APR + RDV, the lgTCID50/mL value was lower by 1.33 and 0.74 than in the group of untreated animals. However, under the conditions studied, in groups FVP and APR + FVP, the lgTCID50/mL values were almost the same (7.33 and 7.43, respectively) and, in group APR + RDV, the lgTCID50/mL values were even higher (7.03) than in the comparison group (6,37). 

Due to the short observation period (4 days), it is difficult to reliably interpret the change in the body weight of animals infected with SARS-CoV-2 and treated (Figure 2). However, it can be noted that these changes are consistent with the above data on the virus titer in the lungs of animals.

## 4. Materials and Methods

### 4.1. Drugs

The following antiviral drugs and their combinations were used in the work: APR with an activity of 5400 KIU/mg from Wanhua Biochem (Nanchang, China), MOV from Jiangsu Zenji Pharmaceuticals Ltd. (Huaian, China), NMV from Shanghai XingMo Biotechnology Co. Ltd. (Shanghai, China), FVP from Zenji Pharmaceuticals (Suzhou) Ltd. (Suzhou, China), RDV from Zenji Pharmaceuticals (Suzhou) Ltd. (Suzhou, China), APR + MOV, APR + NMV, APR + FVP, 1and APR + RDV. Table 3 shows methods for preparing medicines for intraperitoneal administration to mice and drug doses. The prepared medicines were stored at room temperature and in the dark for no more than 8 h.

### 4.2. Mice

In the model of influenza pneumonia, BALB/c mice females weighing 12–14 g were infected with the influenza A/CA/04/2009 (H1N1) virus. The mice were obtained from the animal facility Stezar (Vladimir, Russia).

In the model of COVID-19, BALB/c female transgenic 6–8 weeks old mice of line B6.Cg-Tg(K18-ACE2)2Prlmn/HEMI hemizygous for Tg(K18-ACE2)2Prlmn weighing 19–24 g were infected with SARS-CoV-2. The mice were obtained from Jackson Immunoresearch (West Grove, PA, USA).

More details on mice handling in the study are available in Supplementary Materials Section S1.

### 4.3. Virus and Cell Lines

In the model of influenza pneumonia in mice, the influenza A/CA/04/2009 (pnm H1N1 2009) virus obtained from WHO and adapted to mice was used to infect animals. To prepare the infecting material, three mice were infected intranasally with an allantoic virus, and, after the manifestation of signs of the disease, they were euthanized, and a lung tissue homogenate was obtained under sterile conditions. Further, this homogenate was used to infect 9-day-old chicken embryos, from which the allantoic virus was obtained, and after titration in mice, it was used to infect animals. In the experiments, a pool of virus was used, which was obtained in October 2021, and aliquots were stored at −80 °C. For virus titration, a culture of MDCK cells (transplantable dog kidney cells) obtained from WHO (storage of cultures in liquid nitrogen) was used. The work with the specified virus was carried out in the conditions of laminar boxes of the BSL2 facilities using disposable consumables.

In a model of COVID-19 in transgenic mice, we used a pool of laboratory strain SARS-CoV-2 “Dubrovka” strain (identification number GenBank: MW161041.1), passaged with Vero CCL8 obtained in December 2020. The determination of the infectious titer of the SARS-CoV-2 virus in the Vero CCL81 cell culture showed that it was 107.5 × TCID50/0.1 mL. 

To determine the dose of virus to infect mice, they were challenged with the following dilutions of the SARS-CoV-2 virus: whole, 1:10, 1:20, 1:50, and 1:100, 30 µL in each nostril under light anesthesia. On the 4th day after infection in each group, one animal was euthanized, and its lungs and brain were taken for the determination of virus content.

The data on observation of animals for 7 days are presented in Table 4. From these data, 100% death of the animals was observed in all groups; however, in the group of animals infected with the SARS-CoV-2 virus at a dilution of 1:100, it was delayed up to 7 days, while in other groups the animals died 4–5 days after infection. Determination of the virus titer in the lungs in the Vero CCL81 cell culture also showed that, in all groups, it exceeds 6 lgTCID50/mL. In the lungs of mice that died as a result of infection, a high concentration of viral RNA, more than 9 lg RNA copies/mL of homogenate, was detected by quantitative RT–PCR which is consistent with the virus titration data. Taking into account the obtained data and literature reports on determining the lethal dose in mice, we chose an infecting dose of 103.5 × TCID50 (1:1000 dilution) for infection with SARS-CoV-2.

### 4.4. Study Design

#### 4.4.1. Study Design of the Combinatorial Use of APR and Inhibitors of RNA Viruses in a Model of Influenza Pneumonia in Mice

In the experiment, 10 groups of BALB/c female mice were formed, 13 animals per group, of which 10 mice were tested for survival and 3 mice were tested for the virus titer in lungs (lgTCID50/mL).

Mice were randomized into groups and then were infected intranasally with influenza A/CA/04/2009 (H1N1) virus under light anesthesia at a dose of 5MLD50/mL (25 μL in each nostril—104.5TCID50/mL).

Mice were treated according to the following scheme: in the morning of day 1, the drug was administered to mice intraperitoneally immediately after the virus infection and in the evening (~8–12 h after infection); days 2–5: treatment two times a day. Mice survival was monitored for 16 days. The measurement of the virus titer in the lungs was carried out on the 6th day after the last administration of the drug.

Obtaining mouse lung samples for the study and the determination of viral titer were performed as described in Supplementary Materials Section S2.

The activity of the compounds in the model of influenza pneumonia in mice was evaluated according to the following criteria: animal survival, increase in average life expectancy, dynamics of weight loss, and decrease in virus titer in the lungs of animals on the 6th day after infection.

The percentage of mortality was determined as the ratio of the number of dead animals to the total number of infected animals in the group. The average life expectancy of animals was determined from the calculation of the total number of days of observation of animals (after infection) according to the formula: MSD = ∑f(d − 1)/n, where f is the number of mice that died on day d, and the surviving mice are also included in f and d; in this case, n, is the number of mice in the group [51].

Mice were weighed before the administration of the test substances every other day. The decrease or increase in weight was calculated separately for each mouse and expressed as a percentage. In this case, the weight of the animal before infection was taken as 100%. For all mice in one group, the average value of the percentage of weight loss or weight gain was determined.

The criterion for the antiviral effect was considered to be a statistically significant increase in the survival of animals (*p* < 0.05), an increase in their life expectancy and a statistically significant decrease in the viral titer in the lungs of infected animals (on average > 1.75 lgTCID50) after the administration of experimental samples compared to the control group of infected and untreated animals. The obtained data were subjected to statistical processing in the Statistica 8.0” software. The comparison of survival in groups of mice was performed using a one-way analysis of variance (ANOVA) in “Statistica 8” software.

#### 4.4.2. Study Design of the Combinatorial Use of APR and Inhibitors of RNA Viruses in a Model of Coronavirus Infection Caused by SARS-CoV-2 in Transgenic Mice

In the experiment, 10 groups of transgenic female mice with 4 animals per group were formed. The effectiveness of APR + MOV, APR + NMV, APR + FVP, and APR + RDV combinations were studied in parallel with the control group (saline) and comparison single-component groups: APR, MOV, NMV, FVP, and RDV (Figure 3 and Figure 4). 

The treatment regimen for transgenic mice was as follows: parenteral (intraperitoneal) administration of drugs 2 times a day; day 0–1 h before infection with mouse-adapted SARS-CoV-2 and 6–8 h after infection; days 1, 2, 3—2 times a day, for a total of 4 days (days 0, 1, 2, 3); day 4—lung sampling from all animals to assess the virus titer in the lungs, visual assessment of the lungs, and the transfer of the lungs for histology; days 0–4—daily assessment of the body weight and condition of mice. 

On day 0, animals from all groups were infected with the SARS-CoV-2 “Dubrovka” strain (103.5 TCID50/mL). All mice were infected intranasally under light ether anesthesia in a volume of 30 µL for both nostrils.

On day 4 post infection with the virus, the animals in each group were sacrificed, and the lungs were removed under sterile conditions. One lung was fixed in formalin for further histology, and the second lung was prepared to measure the virus titer. Obtaining mouse lung samples for study and the determination of the viral titer was performed as described in Supplementary Materials Section S2.

The obtained data were statistically processed using Statistica 8.0 software. 

The results are presented in Table 2 and in Figure 4, from which it follows that the APR + MOV and APR + NMV combinations are more effective than their single components in reducing the virus titer in the lungs and the dynamics of body weight loss after 5 days, while the effectiveness of combinatorial preparations APR + FVP and APR + RDV is comparable with their constituent components.

## 5. Conclusions

Based on the data, we can conclude that combination treatment with APR and antiviral drugs improved outcomes in mice infected with influenza virus and SARS-CoV-2, as it drastically reduced the virus titer value in the lungs of animals. Apparently, with a high probability, this is due to the therapeutic profile of aprotinin and, above all, its anti-inflammatory and antithrombotic properties.

These results represent the next promising step for the clinical evaluation of combination therapy with APR and antiviral drugs (RDV, MOV, FVP and NMV) in diseases caused by influenza viruses and coronaviruses, in particular pneumonia.

## Figures and Tables

**Table 1:** Efficacy of drugs in the mouse influenza pneumonia model for infection with mouse-adapted influenza A/CA/04/2009 (H1N1) virus.

Group			lgTCID_50_/mL *^b^*	Life *^c^*		
No.	Drug *^a^*	Dose, mg/kg		Alive *^c^* (*p*)	Mortality, %	Average Life Expectancy, Days
1	Saline		>7	0	100	7.6
2	APR	50,000 *^d^*	4.75 ± 0.43	3 (0.0652)	70	10.7
3	RDV	5	4.33 ± 0.76	4 (0.0248)	60	10.8
4	MOV	5	4.17 ± 0.58	5 (0.0077)	50	11.4
5	FVP	5	2.67 ± 0.29	6 (0.0017)	40	12.4
6	AV5080	0.25	3.92 ± 0.8	7 (0.0002)	30	13.0
7	APR + RDV	50,000 *^d^* + 5	3.5 ± 0	5 (0.0077)	50	10.4
8	APR + MOV	50,000 *^d^* + 5	3.5 ± 0.66	5 (0.0077)	50	11.8
9	APR + FVP	50,000 *^d^* + 5	2.08 ± 0.14	9	0	16.0
10	APR + AV5080	50,000 *^d^* + 0.25	2.5 ± 0.87	8 (0.00001)	20	14.0

*^a^* Group No. 1- Control, intragastric (IG) administration (ADMIN) saline; groups 2, 7, 8, 9, and 10 intraperitoneal ADMIN of APR; groups 3, 4, 5, 6, 7, 8, 9, and 10 IG ADMIN of AV5080, FVP, MOV, and RDV respectively. *^b^* A titer value of the virus in the mouse lungs 5 days after infection. *^c^* Out of 10 animals; *p*-value ≤ 0.05 is statistically significant. *^d^* KIU/kg.

**Table 2:** Efficacy endpoints in the mice model of COVID-19.

Median Time, Days (IQR)	Cohorta			
	1 *^a^*	2 *^b^*	3 *^c^*	4 *^d^*
to the elimination of SARS-CoV-2	3.5 (3–4)	7.5 (6–9)	4.5 (4–9)	9.0 (5–9)
to the normalization of CRP	3.5 (3–5)	6.0 (6–6)	14.0 (5.5–14)	14.0 (14–14)
to the normalization of D-dimer	5.0 (4–5)	4.5 (3–6)	NA	NA
to the normalization of body temp.	1.0 (1–3)	3.0 (2–3)	2.0 (1–3)	4.0 (1–8)
to an improvement in clinical status by 2 points on the WHO-OSCI	5.0 (5–5)	11.0 (6–11)	14.0 (11.5–16)	13.0 (11.5–15.5)

*^a^* (1): IV APR + PO avifavir (FVP) + standard of care (SOC). *^b^* (2): IV APR + PO hydroxychloroquine (HCQ) + SOC. *^c^* (3): PO avifavir (FVP) + SOC. *^d^* (4): PO HCQ + SOC.

**Table 3:** Methods of medicine preparation for intraperitoneal administration to mice and drug doses in the mouse influenza pneumonia model and in a model of coronavirus infection caused.

No.	Drug	Preparing of Medicines	Conc.	Daily Dose for 2.1
1	Saline	sodium chloride in distilled water	0.9%	5 mL/kg
2	APR	A total of 1852 mg APR (5400 KIU/mg) in 1 mL of saline is stirred with ultrasound until complete dissolution.	10,000 KIU/mL	50,000 KIU/kg
3	AV5080	A total of 0.05 mg AV5080 in 1 mL of saline is stirred with ultrasound until complete dissolution.	0.05 mg/mL	0.25 mg/kg
4	FVP	A total of 50 mg FVP and 24 mg L-lysine monohydrate in 1 mL of saline are stirred with ultrasound until complete dissolution before the addition of ~25 µL 10 N NaOH; it is then stirred with ultrasound until complete dissolution and pH 7–8 are achieved.	50 mg/mL	250 mg/kg
5	MPV	A total of 5 mg MPV in 1 mL of saline is stirred with ultrasound until complete dissolution.	5 mg/mL	25 mg/kg
6	RDV	A total of 5 mg RDV in 1 mL of saline is stirred with ultrasound until complete dissolution.	5 mg/mL	25 mg/kg
7	NMV	A total of 20 mg NMV in 1 mL of saline is stirred with ultrasound until complete dissolution.	20 mg/mL	100 mg/kg
8	APR + AV5080	A total of 1 mL APR (10,000 KIU/mL) and 0.05 mg AV5080 are stirred with ultrasound until complete dissolution.	0.05 mg/mL	50,000 KIU/kg + 0.25 mg/kg
9	APR + FVP	A total of 1 mL APR (10,000 KIU/mL) and 50 mg FVP + 24 mg L-lysine monohydrate are stirred with ultrasound until complete dissolution before the addition of ~25 µL 10 N NaOH; it is then stirred with ultrasound until complete dissolution and pH 7–8 are achieved.	10,000 KIU/mL + 50 mg/mL	50,000 KIU/kg + 250 mg/kg
10	APR + MPV	A total of 1 mL APR (10,000 KIU/mL) and 5 mg MPV are stirred with ultrasound until complete dissolution.	10,000 KIU/mL + 5 mg/mL	50,000 KIU/kg + 25 mg/kg
11	APR + RDV	A total of 1 mL APR (10,000 KIU/mL), 5 mg RDV and 300 mg SBECD are stirred with ultrasound until complete dissolution.	10,000 KIU/mL + 5 mg/mL	50,000 KIU /kg + 25 mg/kg
12	APR + NMV	A total of 1 mL APR (10,000 KIU/mL) and 20 mg NMV are stirred with ultrasound until complete dissolution.	10,000 KIU/mL + 20 mg/mL	50,000 KIU /kg + 100 mg/kg

**Table 4:** Determination (MLD) of the minimum lethal dose (MLD50) in mice infected with the SARS-CoV-2 Vero «Dubrovka» virus.

Virus Breeding	Survivors/Dead	Mortality, %	Virus Titer in the Lungs, lg TCD_50_/mL	Conc. of Viral RNA in Lungs, lg RNA Copies/mL
Whole	0/2	100	>6	9.3
1:10	0/2	100	>6	10.3
1:20	0/2	100	>6	9.7
1:50	0/2	100	>6	10.3
1:100	0/2 *^a^*	100	>6	NA *^b^*

*^a^* Delayed death. *^b^* Not analyzed.

## Data Availability

Supporting data are provided as Supplementary Materials.

## Supplementary Materials

The following supporting information can be downloaded at: https://www.mdpi.com/article/10.3390/molecules27154975/s1. Supplementary Materials contain protocols about a model of influenza pneumonia in mice and obtaining mouse lung samples for study and determination of viral titer.

## References

[1] Cucinotta D., Vanelli M. (2020). WHO Declares COVID-19 a Pandemic. Acta Biomed..

[2] (2022). COVID-19 Coronavirus Pandemic. Worldometer. https://www.worldometers.info/coronavirus.

[3] Qomara W.F., Primanissa D.N., Amalia S.H., Purwadi F.V., Zakiyah N. (2021). Effectiveness of Remdesivir, Lopinavir/Ritonavir, and Favipiravir for COVID-19 Treatment: A Systematic Review. Int. J. Gen. Med..

[4] Masyeni S., Iqhrammullah M., Frediansyah A., Nainu F., Tallei T., Emran T.B., Ophinni Y., Dhama K., Harapan H. (2022). Molnupiravir: A lethal mutagenic drug against rapidly mutating severe acute respiratory syndrome coronavirus 2—A narrative review. J. Med. Virol..

[5] Javanian M., Barary M., Ghebrehewet S., Koppolu V., Vasigala V.K.R., Ebrahimpour S. (2021). A brief review of influenza virus infection. J. Med. Virol..

[6] CDC, NCIRD (2021). Disease Burden of Flu. https://www.cdc.gov/flu/about/burden/index.html.

[7] CDC (2021). Archived: Estimated Influenza Illnesses, Medical Visits, Hospitalizations, and Deaths in the United States—2019–2020 Influenza Season. https://www.cdc.gov/flu/about/burden/2019-2020/archive-09292021.html.

[8] CDC (2022). Influenza Antiviral Medications: Summary for Clinicians. https://www.cdc.gov/flu/professionals/antivirals/summary-clinicians.htm.

[9] Bai Y., Jones J.C., Wong S.-S., Zanin M. (2021). Antivirals Targeting the Surface Glycoproteins of Influenza Virus: Mechanisms of Action and Resistance. Viruses.

[10] Louie J.K., Yang S., Acosta M., Yen C., Samuel M.C., Schechter R., Guevara H., Timothy Uyeki M. (2012). Treatment with neuraminidase inhibitors for critically ill patients with nfluenza A (H1N1) pdmClin. Infect. Dis..

[11] Ivachtchenko A.V., Ivanenkov Y.A., Mitkin O.D., Yamanushkin P.M., Bichko V.V., Shevkun N.A., Karapetian R.N., Leneva I.A., Borisova O.V., Veselov M.S. (2014). Novel oral anti-influenza drug candidate AV5080. J. Antimicrob. Chemother..

[12] Bojkova D., Bechtel M., McLaughlin K.M., McGreig J.E., Klann K., Bellinghausen C., Rohde G., Jonigk D., Braubach P., Ciesek S. (2020). Aprotinin Inhibits SARS-CoV-2 Replication. Cells.

[13] Scheule A.M., Beierlein W., Wendel H.P., Jurmann M.J., Eckstein F.S., Ziemer G. (1999). Aprotinin in fibrin tissue adhesives induces. specific antibody response and increases antibody response of high-dose intravenous application. J. Thorac. Cardiovasc. Surg..

[14] Bidstrup B.P., Royston D., Sapsford R.N., Taylor K.M. (1989). Reduction in blood loss and blood use after cardiopulmonary bypass with high dose aprotinin (Trasylol). J. Thorac. Cardiovasc. Surg..

[15] Royston D. (1996). Preventing the inflammatory response to open-heart surgery: The role of aprotinin and other protease inhibitors. Int. J. Cardiol..

[16] Lentschener C., Benhamou D., Mercier F.J., Boyer-Neumann C., Naveau S., Smadja C., Wolf M., Franco D. (1997). Aprotinin reduces blood loss in patients undergoing elective liver resection Affiliations expand. Anesth. Analg..

[17] Zhirnov O.P., Ovcharenko A.V., Bukrinskaya A.G. (1982). A modified plaque assay method for accurate analysis of infectivity of influenza viruses with uncleaved hemagglutinin. Arch. Virol..

[18] Zhirnov O.P., Ovcharenko A.V., Bukrinskaya A.G. (1984). Suppression of Influenza Virus Replication in Infected Mice by Protease lnhibitors. J. Gen. Virol..

[19] Zhirnov O.P., Klenk H.D., Wright P.F. (2011). Aprotinin and similar protease inhibitors as drugs against influenza. Antivir. Res..

[20] Shen L.W., Mao H.J., Wu Y.L., Tanaka Y., Zhang W. (2017). TMPRSS2: A potential target for treatment of influenza virus and coronavirus infections. Biochimie.

[21] Lambertz R.L.O., Gerhauser I., Nehlmeier I., Gärtner S., Winkler M., Leist S.R., Kollmus H., Pöhlmann S., Schughart K. (2020). H2 influenza A virus is not pathogenic in Tmprss2 knock-out mice. Virol. J..

[22] Hoffmann M., Kleine-Weber H., Schroeder S., Krüger N., Herrler T., Erichsen S., Schiergens T.S., Herrler G., Wu N.H., Nitsche A. (2020). SARS-CoV-2 Cell Entry Depends on ACE2 and TMPRSS2 and is Blocked by a Clinically Proven Protease Inhibitor. Cell.

[23] Hoffmann M., Schroeder S., Kleine-Weber H., Müller M.A., Drosten C., Pöhlmann S. (2020). Nafamostat mesylate blocks activation of SARS-CoV-2: New treatment option for COVID-Antimicrob. Agents Chemother..

[24] Meng B., Abdullahi A., Ferreira I.A.T.M., Goonawardane N., Saito A., Kimura I., Yamasoba D., Gerber P.P., Fatihi S., Rathore S. (2022). Altered TMPRSS2 usage by SARS-CoV-2 Omicron impacts infectivity and fusogenicity. Nature.

[25] Bojkova D., Widera M., Ciesek S., Wass M.N., Michaelis M., Cinatl J. (2022). Reduced interferon antagonism but similar drug sensitivity in Omicron variant compared to Delta variant of SARS-CoV-2 isolates. Cell Res..

[26] Limburg H., Harbig A., Bestle D., Stein D.A., Moulton H.M., Jaege J., Janga H., Hardes K., Koepke J., Schulte L. (2019). TMPRSS2 Is the Major Activating Protease of Influenza A Virus in Primary Human Airway Cells and Influenza B Virus in Human Type II Pneumocytes. J. Virol..

[27] Wettstein L., Kirchhoff F., Münch J. (2022). The Transmembrane Protease TMPRSS2 as a Therapeutic Target for COVID-19 Treatment. Int. J. Mol. Sci..

[28] Bestle D., Heindl M.R., Limburg H., van Lam T.V., Pilgram O., Moulton H., Stein D.A., Hardes K., Eickmann M., Dolnik O. (2020). TMPRSS2 and furin are both essential for proteolytic activation of SARS-CoV-2 in human airway cells. Life Sci. Alliance.

[29] Rahbar S.Y., Hosseiniyan K.S.M., Zununi V.S., Ardalan M. (2021). Host Serine Proteases: A Potential Targeted Therapy for COVID-19 and Influenza. Front. Mol. Biosci..

[30] Zhirnov O.P., Golyando P.B., Ovcharenko A.V. (1994). Replication of influenza B virus in chicken embryos is suppressed by exogenous aprotinin. Arch. Virol..

[31] Zhirnov O.P., Bokova N.O., Isaeva E.I., Vorobieva I.V., Malyshev N.A. (2015). Pathogenetic treatment of influenza patients with aerosolized form of aprotinin, a protease inhibitor. BIOpreparations. Prev. Diagn. Treat..

[32] Bertram S., Glowacka I., Steffen I., Kühl A., Pöhlmann S. (2010). Novel insights into proteolytic cleavage of influenza virus hemagglutinin. Rev. Med. Virol..

[33] Böttcher E., Tatyana Matrosovich T., Beyerle M., Klenk H.-D., Garten W., Matrosovich M. (2006). Proteolytic activation of influenza viruses by serine proteases TMPRSS2 and HAT from human airway epithelium. J. Virol..

[34] Bertram S., Heurich A., Lavender H., Gierer S., Danisch S., Perin P., Lucas J.M., Nelson P.S., Pöhlmann S., Soilleux E.J. (2012). Influenza and SARS-coronavirus activating proteases TMPRSS2 and HAT are expressed at multiple sites in human respiratory and gastrointestinal tracts. PLoS ONE.

[35] Song E.-J., Españo E., Shim S.-M., Nam J.-H., Kim J., Lee K., Park S.-K., Lee C.-K., Kim J.-K. (2021). Inhibitory effects of aprotinin on influenza A and B viruses in vitro and in vivo. Sci. Rep..

[36] Zhirnov O.P., Matrosovich T.Y., Matrosovich M.N., Klenk H.-D. (2011). Aprotinin, a protease inhibitor, suppresses proteolytic activation of pandemic H1N1v influenza virus. Antivir. Chem. Chemother..

[37] Zhirnov O.P., Ikizler M.R., Wright P.F. (2002). Cleavage of influenza A virus hemagglutinin in human respiratory epithelium is cell associated and sensitive to exogenous antiproteases. J. Virol..

[38] Zhirnov O.P., Khanykov A.V. (2010). Aprotinin-Based Aerosol Preparation for the Treatment of Viral Respiratory Infections.

[39] Furuta Y., Takahashi K., Fukuda Y., Kuno M., Kamiyama T., Kozaki K., Nomura N., Egawa H., Minami S., Watanabe Y. (2002). In Vitro and In Vivo Activities of Anti-Influenza Virus Compound T-Antimicrob. Agents Chemother..

[40] Furuta Y., Komeno T., Nakamura T. (2017). Favipiravir (T-705), a broad spectrum inhibitor of viral RNA polymerase. Proc. Jpn. Acad. Ser. B Phys. Biol. Sci..

[41] Yoon J.J., Toots M., Lee S., Lee M.-E., Ludeke B., Luczo J.M., Ganti K., Cox R.M., Sticher Z.M., Edpuganti V. (2018). Orally efficacious broad-spectrum ribonucleoside analog inhibitor of influenza and respiratory syncytial viruses. Antimicrob. Agents Chemother..

[42] Toots M., Yoon J.J., Cox R.M., Hart M., Sticher Z.M., Makhsous N., Plesker R., Barrena A.H., Reddy P.G., Mitchell D.G. (2019). Characterization of orally efficacious influenza drug with high resistance barrier in ferrets and human airway epithelia. Sci. Transl. Med..

[43] Toots M., Yoon J.J., Hart M., Natchus M.G., Painter G.R., Plemper R.K. (2020). Quantitative efficacy paradigms of the influenza clinical drug candidate EIDD-2801 in the ferret model. Transl. Res..

[44] Stephens B. The Story of Remdesivir. The New York Times. https://www.nytimes.com/2020/04/17/opinion/remdesivir-coronavirus.html.

[45] (2020). FDA Approves First Treatment for COVID-FDA. https://www.fda.gov/news-events/press-announcements/fda-approves-first-treatment-covid-19.

[46] NIH (2021). Influenza and COVID—COVID-19 Treatment Guidelines. https://www.covid19treatmentguidelines.nih.gov/special-populations/influenza/#:~:text=Remdesivir%20does%20not%20have%20activity,who%20are%20receiving%20oseltamivir%20treatment.

[47] Alsafi R., Alghamdi S., Asif M. (2022). Antiviral Drugs and Their Roles in the Treatment of Coronavirus. Antiviral Drugs.

[48] Wang D., Hu B., Hu C., Zhu F., Liu X., Zhang J., Wang B., Xiang H., Cheng Z., Xiong Y. (2020). Clinical Characteristics of 138 Hospitalized Patients With 2019 Novel Coronavirus–Infected Pneumonia in Wuhan, China. JAMA.

[49] Ivashchenko A.A., Azarova V.N., Egorova A.N., Karapetian R.N., Kravchenko D.V., Krivonos N.V., Loginov V.G., Poyarkov S.V., Merkulova E.A., Rosinkova O.S. (2021). Effect of Aprotinin and Avifavir^®^ Combination Therapy for Moderate COVID-19 Patients. Viruses.

[50] Redondo-Calvo F.J., Padín J.F., Muñoz-Rodríguez J.R., Serrano-Oviedo L., López-Juárez P., Porras Leal M.L., González Gasca F.J., Rodríguez Martínez M., Pérez Serrano R., Sánchez Cadena A. (2022). Aprotinin treatment against SARS-CoV-2: A randomized phase III study to evaluate the safety and efficacy of a pan- protease inhibitor for moderate COVID-19. Eur. J. Clin. Investig..

[51] Leneva I., Roberts N., Govorkova E., Goloubeva O., Webster R.G. (2000). The neuraminidase inhibitor GS4104 (oseltamivir phosphate) is efficacious against A/Hong Kong/156/97 (H5N1) and A/Hong Kong/1074/99 (H9N2) influenza virus. Antivir. Res..

