# Supplementary material for: The Efficacy of Aprotinin Combinations with Selected Antiviral Drugs in Mouse Models of Influenza Pneumonia and Coronavirus Infection Caused by SARS-CoV-2
Source: Molecules. 2022 Aug 5;27(15):4975. doi: 10.3390/molecules27154975 (PMC9370800; doi:10.3390/molecules27154975)
Supplement: Supplementary file 1 [file molecules-27-04975-s001.zip › molecules-1813069-supplementary.pdf]

Supplementary Materials

1. Mice in a model of influenza pneumonia in mice.

The mice were kept in the vivarium under additional quarantine for 3 days. When forming the experimental groups, each animal was assigned an individual number in each group. Animals were marked with eosin staining. On the card of the cage in which the animals were kept, the name of the study, the group number, and the administered substance were indicated.

The animals were kept in accordance with the rules adopted by the European Convention for the Protection of Vertebrate Animals used for Experimental and Other Scientific Purposes (Strasbourg, 1986).

Mice were housed in 4–5 individual plastic cages with sterilized small chips as bedding in accordance with the rules of placement. In the room where the animals were kept, ambient temperature of 18–26 °C and relative humidity of 30–70% were maintained; the automatic change of a 12-h light period (08:00–20:00 - day, 20:00–08:00 - night) and ventilation without recirculation with air change of 7–12 room volumes per hour were applied. The animals were fed with briquetted feed in accordance with approved standards. Animals were fed with distilled water. The access of animals to water and food was unlimited.

Mice adapted to the vivarium of the institute in a separate room for 3 days before the introduction of the analyzed samples. During this period, daily physical examination of the animals and clinical examination prior to randomization were performed. Animals with abnormalities were not identified during the inspection.

Euthanasia (painless killing of the animal) was performed by a responsible person through the dislocation of the cervical vertebrae with preliminary anesthesia with ether. Euthanasia was carried out in a timely manner at the end of the experiments.

2. Obtaining mouse lung samples for study and determination of viral titer.

On the 6th day after infection with the influenza virus, three mice were euthanized in each group, and their lungs were removed under sterile conditions, then homogenized and finally resuspended in 1 mL of cold sterile 0.01 M PBS. The suspension was cleared of cell debris by centrifugation at 2000 g for 10 min. Then 0.1 mL of the supernatant was used to determine the infectious titer of the virus. To determine the infectious titer of the virus, the MDCK cells were seeded in 96-well plates from Costar with an average density of 30000–35000 cells per well and grown in Eagle's minimum medium (MEM) in the presence of 5% fetal calf serum, 10 mM glutamine, and antibiotics (penicillin 100 IU/mL and streptomycin 100 μg/mL) until there was a complete monolayer. Before infection with the virus, the cells were washed twice with MEM medium without serum. Ten-fold dilutions of each virus sample from the lungs (whole to 10-8) were prepared on a medium supplemented with TRNC-trypsin (2 μg/mL). The obtained dilutions infected the monolayer of four wells in a 96-well plate. After incubation at 37°C in the atmosphere of 5% CO_2 for 72 h, the cells were washed three times with PBS and fixed with 10% formaldehyde solution at a temperature of 18–23°C for 5 min. After the formaldehyde solution was removed, 100 μL of a 1% crystal violet solution was added to each well of the plate and kept at a temperature of 18–23 ºC for 5 min. After washing with water and drying the plate, 0.1 mL of 96% alcohol was added to the wells and incubated with shaking at room temperature for 20 min, and then the optical density was measured at a wavelength of 570 nm. The wells were considered positive if the optical density in them was less than the optical density in the cell control by 20%. The infectious titer of the virus was determined from four replicates of each sample according to the method of Reed and Mench and expressed in $lgTCID_{50}$/mL (tissue cytopathic infectious dose 50). Then the average titer value for five identical samples was calculated (Table 1).
